# Fatal X-linked agammaglobulinemia complicated by septic shock: a case report and comprehensive review of novel BTK mutations

**DOI:** 10.3389/fimmu.2025.1645337

**Published:** 2025-08-15

**Authors:** Piaopiao Wu, Wei You, Wentong Liu, Ni Luo, Jiayue Xu

**Affiliations:** ^1^ Department of Gerontology, CR & WISCO General Hospital Affiliated to Wuhan University of Science and T echnology, Wuhan, Hubei, China; ^2^ School of Basic Medicine, Hubei University of Arts and Science, Xiangyang, China

**Keywords:** case report, BTK mutations, septic shock, delayed diagnosis, primary immunodeficiency

## Abstract

X-linked agammaglobulinemia (XLA) is a rare primary immunodeficiency disorder caused by mutations in the Bruton tyrosine kinase (BTK) gene. This article presents a fatal case of a 20-year-old male with XLA complicated by septic shock due to *Pseudomonas aeruginosa* infection, highlighting two novel BTK insertion mutations in exon 15 (NM_000061.3 exon15:c.1561insG and c.1565insTAGAA). Concurrently, we provide a systematic review of XLA’s genetic basis, clinical manifestations, diagnostic challenges, and therapeutic advancements. The patient’s delayed diagnosis, lack of immunoglobulin replacement therapy, and fatal outcome underscore the importance of early genetic screening and standardized management. This case and review aim to enhance clinical awareness and emphasize the integration of genetic diagnostics into routine practice for primary immunodeficiencies.

## Introduction

X-linked agammaglobulinemia (XLA) is a rare primary immunodeficiency disorder (PID) (1, 2). It was first described in 1952 by the American pediatrician Ogden Bruton, making it the first hereditary immunodeficiency identified in humans (3, 4). The disease is caused by mutations in the Bruton’s tyrosine kinase (BTK) gene located on the X chromosome (Xq22), leading to B-cell developmental arrest (5). This results in a significant reduction or absence of mature B cells and extremely low or undetectable levels of immunoglobulins (IgG, IgA, IgM) (6).

Affected individuals are typically male (females are usually carriers), and symptoms usually appear 6–12 months after birth, when maternal antibody protection wanes. Patients suffer from recurrent bacterial infections (e.g., otitis media, pneumonia, sinusitis), particularly from encapsulated bacteria (e.g., Streptococcus pneumoniae, Haemophilus influenzae) (7). Without timely treatment, complications such as chronic lung disease and sepsis may occur. Intravenous immunoglobulin (IVIG) replacement therapy and antibiotic prophylaxis are the mainstays of management. Advances in genetic diagnosis and early intervention have significantly improved patient outcomes, but lifelong monitoring is required.

In this study, we report a previously undocumented BTK mutation in an XLA patient who died from septic shock and multi-organ failure.

## Case presentation

### Chief complaints

A 20-year-old male was admitted with fever (peak temperature: 39°C), cough, bloody sputum, and hemoptysis for two days. He was initially diagnosed with septic shock and treated with antibiotics and vasopressors, but his condition did not improve. He was subsequently transferred to the ICU of a tertiary hospital.

### History of past illness

The patient had a 7-year history of bronchiectasis and had been hospitalized multiple times in the past two years for severe pneumonia. He denied any history of hypertension, diabetes, coronary heart disease, hepatitis, tuberculosis, trauma, blood transfusions, or surgeries. He also had no history of long-term smoking, alcohol abuse, or drug allergies.

### Personal and family history

His mother denied any specific family history.

### Physical examination

On admission, his temperature was 38.7°C, heart rate 148 bpm, respiratory rate 39 breaths/min, and blood pressure 94/52 mmHg (maintained with vasopressors). Oxygen saturation was 94% (with oxygen support). The patient was conscious but appeared acutely ill and lethargic. Coarse breath sounds and dry rales were heard in both lungs. A scattered rash was present on his back and buttocks, with a stage II pressure ulcer (6 cm × 3.5 cm) on the left ischial tuberosity. The left lower limb showed scattered eczema and ulcerations. No lymphadenopathy was detected in the neck or supraclavicular fossae. There was no jaundice, cyanosis, or edema in the lower limbs.

### Imaging examinations

**Figure 1:**
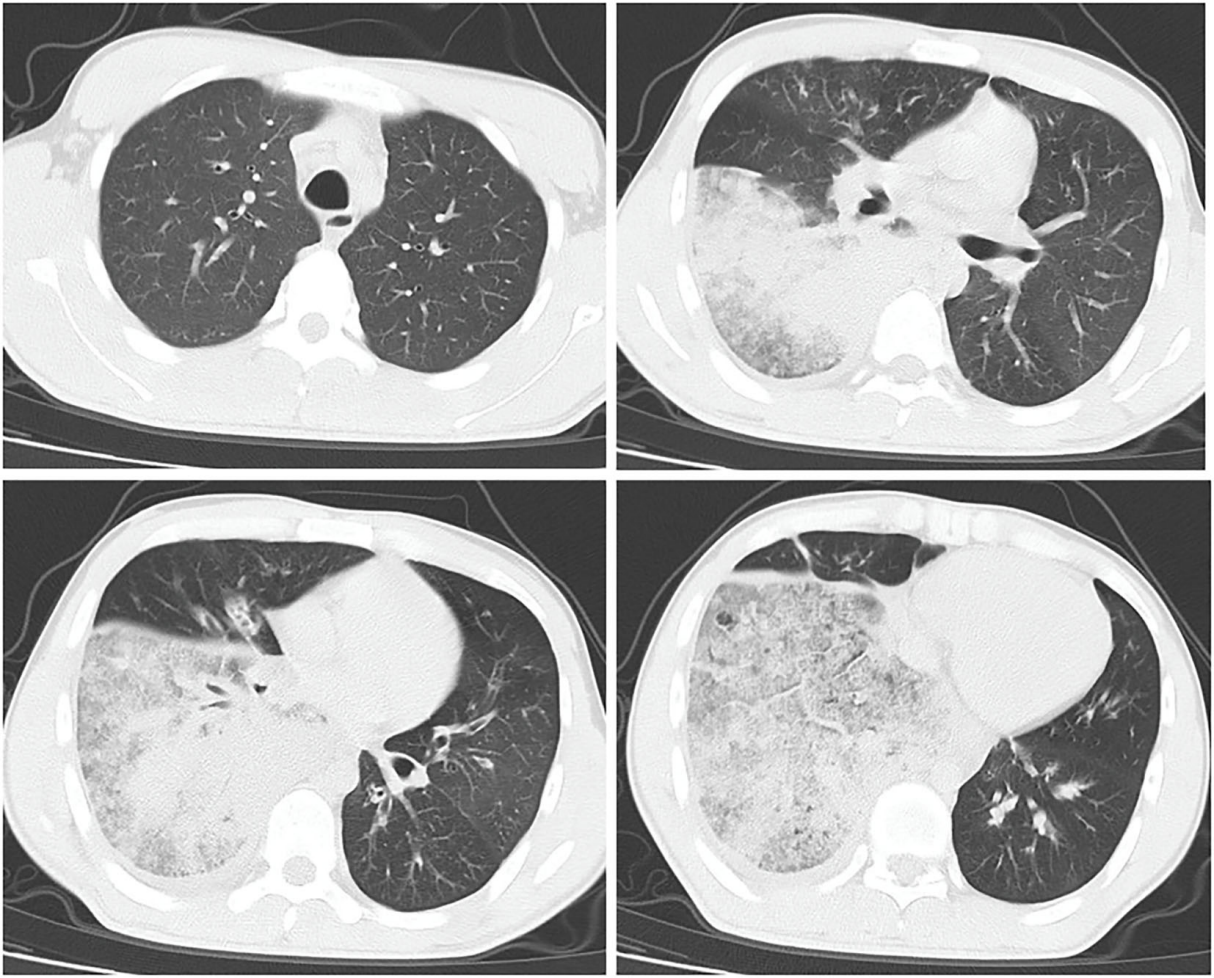
CT results of the lungs.

Chest CT revealed patchy high-density shadows in the left lower lobe and right lung, with significant involvement of the right lower lobe and a small amount of right pleural effusion (**Figure 1**). Bedside thoracic ultrasound confirmed bilateral pleural effusion. Cardiac ultrasound indicated tachycardia, while abdominal, lower limb, and urinary tract ultrasounds showed no abnormalities.

### Laboratory examinations

Initial tests showed elevated white blood cells (12.92 × 10^9^/L) and neutrophils (11.90 × 10^9^/L), suggesting bacterial infection. By the second day, white blood cells surged to 24.98 × 10^9^/L, with neutrophils accounting for 95.7% (23.91 × 10^9^/L) and severe lymphopenia (0.47 × 10^9^/L), indicating severe sepsis. Platelets dropped from 216 × 10^9^/L to 73 × 10^9^/L. Inflammatory markers were markedly elevated: CRP remained extremely high, PCT >200 ng/mL, and a cytokine storm was evident (IL-6 >5000 pg/mL, IL-10 >1000 pg/mL, TNF-α 44.3 pg/mL). Immunoglobulin levels were critically low: IgG <0.3 g/L, IgA <0.07 g/L, IgM <0.04 g/L, with reduced complement C3/C4. Rheumatologic and ANCA tests were negative, excluding autoimmune diseases. Metagenomic next-generation sequencing (mNGS) of bronchoalveolar lavage fluid identified Pseudomonas aeruginosa (**Table 1**).

**Table 1:** NGS detection of alveolar lavage fluid.

Category	Pathogen	Number of sequences	Relative abundance (%)
Bacteria	Pseudomonas aeruginosa	79196	99.0
Fungi	-	-	-
Virus	Parvotitus	18	94.7
Parasite	-	-	-
Specific pathogens	M.Pneumonia	6	0.1
Human microbiome	Veillonelladispar	4	0.1

### Treatment

The patient was immediately treated with antibiotics (meropenem + linezolid), vasopressors (norepinephrine + metaraminol + dopamine), fresh frozen plasma for coagulopathy, IVIG (5 g), and glucocorticoids (dexamethasone). On the second day, he developed respiratory distress with oxygen saturation dropping to 75%, necessitating intubation. Treatment was adjusted to stronger antibiotics (meropenem + tigecycline), increased IVIG (30 g/day), and optimized vasopressor therapy. On the third day, recurrent hypotension and progressive bradycardia culminated in cardiac arrest. Resuscitation efforts failed, and the patient was declared clinically dead.

### Final diagnosis

**Figure 2:**
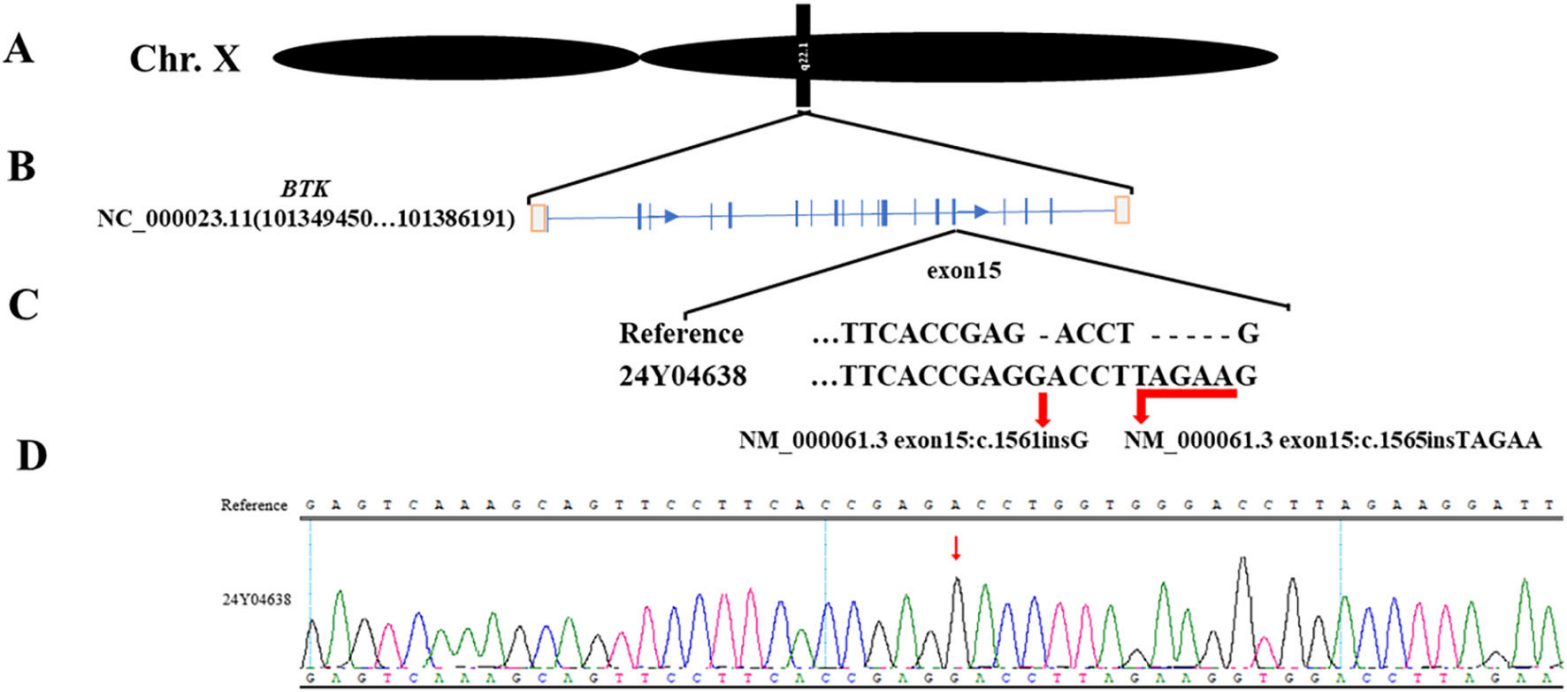
Mutation details. **(A)**. The mutated gene is located on the X chromosome. **(B)**. The two mutation sites are located on exon 15 of the BTK gene. **(C)**. Whole-exome sequencing shows the information of the two mutation sites. **(D)**. Peripheral blood sequencing verifies the mutation results.

With family consent, whole-exome sequencing of peripheral blood cells revealed two previously unreported insertion mutations in exon 15 of the BTK gene on the X chromosome (**Figure 2**). Lymphocyte subset analysis showed near-absence of B cells (**Table 2**), confirming a diagnosis of XLA with a novel BTK mutation. The patient died from Pseudomonas aeruginosa infection leading to multiple organ failure.

**Table 2:** Lymphocyte typing assay.

Inspection Item	Result	Reference Interval	Unit
CD3+CD19-(%)	92.94	50-84	%
CD3+CD19-(#)	472	955-2860	pcs/μL
CD3-CD19+(%)	0.04	5-1860	%
CD3-CD19+(#)	0	90-560	pcs/μL
CD3+CD4+(%)	29.69	27-51	%
CD3+CD4+(#)	151	550-1440	pcs/μL
CD3+CD8+(%)	57.71	15-44	%
CD3+CD8+(#)	293	320-1250	pcs/μL
CD3-/CD16+CD56+(%)	6.19	7-40	%
CD3-/CD16+CD56+(#)	31	150-1100	pcs/μL

## Discussion

XLA is a primary immunodeficiency disorder characterized by impaired B-cell development, leading to antibody deficiency and recurrent infections (8). Patients often present with severe bacterial infections, particularly in the ears, nose, throat, and respiratory tract. A 2022 study of the USIDNET registry involving 231 XLA patients with known BTK mutations identified common pathogenic microorganisms as Haemophilus influenzae (10.4%), *Staphylococcus aureus* (10.4%), Streptococcus pneumoniae (7.8%), and Pseudomonas aeruginosa (6.1%) (9). In this case, the patient had a history of multiple severe pneumonias and was admitted with septic shock, ultimately succumbing to multi-organ failure due to Pseudomonas aeruginosa infection. A 2016 study reported that the average age of onset for XLA was 2.15 ± 2.16 years, with a median of 1 year. Among 135 patients (77.59%), symptoms appeared before the age of 3, and only one case presented at 13 years (10). The average age at diagnosis was 7.09 ± 3.98 years (range: 0.17–19 years), with 87 patients (50.58%) diagnosed within the first 6 years. However, only 5 patients (2.91%) were diagnosed before 1 year of age. This patient exhibited bronchiectasis at 13 but was not properly evaluated. Over the past two years, recurrent pneumonias also failed to prompt an XLA diagnosis. The cornerstone of XLA treatment is regular immunoglobulin replacement therapy to prevent infections (9, 11). Unfortunately, this patient was not diagnosed early or given standardized immunoglobulin therapy, leading to recurrent infections and rapid disease progression. Additionally, septic shock and multi-organ failure further complicated treatment, resulting in a poor prognosis.

Mutations in the BTK gene, are the primary cause of XLA (12). BTK consists of five domains: catalytic kinase (SH1), SH2, Src homology (SH3), Pleckstrin homology (PH), and Tec homology (TH), spanning 19 exons (13, 14). The BTKbase, an international database documenting disease-causing variants in BTK associated with XLA, currently contains information on 2,310 DNA variants from 2,291 individuals, including 1,025 unique variants (15). Mutations predominantly occur in the kinase domain (49.4%), with missense mutations accounting for 41.5% and insertions for 1.8% (https://structure.bmc.lu.se/idbase/BTKbase/). Due to the wide variety of BTK mutations, correlating specific mutations with disease severity remains challenging, and no objective standard for symptom severity exists (16, 17).

In this case, whole-exome sequencing revealed two previously unreported insertion mutations in exon 15 of the BTK gene(NM_000061.3 exon15:c.1561insG, NM_000061.3 exon15:c.1565insTAGAA), located within the catalytic kinase (SH1) domain. These mutations likely impair BTK kinase function, leading to symptom onset at 13 and death at 20 due to Pseudomonas aeruginosa-induced multi-organ failure. Genetic testing is now widely used in clinical practice, and early diagnosis combined with standardized treatment can prevent disease progression (18). Dana O’Toole’s research indicates that respiratory infections are a common cause of death in XLA patients. Screening for B-cell developmental defects in newborns provides an opportunity to prevent pulmonary infections through early diagnosis and treatment initiation. This approach helps increase clinical suspicion of XLA in patients with recurrent infections and assists healthcare providers in anticipating infection patterns in XLA patients (9). Although experimental approaches such as virus-mediated oligonucleotide gene therapy in mice and hematopoietic stem cell gene editing are under investigation, there is currently no cure for XLA in clinical practice (19, 20).

## Conclusion

X-linked agammaglobulinemia is a life-threatening immunodeficiency. Early diagnosis and standardized immunoglobulin replacement therapy are critical. This case underscores the need for heightened clinical awareness of primary immunodeficiencies, particularly in patients with recurrent infections. Prompt immunological and genetic testing can improve outcomes. Therefore, we advocate for the inclusion of severe B-cell developmental defects (such as XLA) in newborn screening programs to prevent such fatal outcomes.

## Data Availability

The original contributions presented in the study are included in the article/supplementary material. Further inquiries can be directed to the corresponding author.
